# Influence of Imperfections on the Effective Stiffness of Multilayer Corrugated Board

**DOI:** 10.3390/ma16031295

**Published:** 2023-02-02

**Authors:** Damian Mrówczyński, Tomasz Garbowski

**Affiliations:** 1 Doctoral School, Department of Biosystems Engineering, Poznan University of Life Sciences, Wojska Polskiego 28, 60-637 Poznań, Poland; 2 Department of Biosystems Engineering, Poznan University of Life Sciences, Wojska Polskiego 50, 60-627 Poznań, Poland

**Keywords:** multilayer corrugated board, numerical homogenization, effective stiffness, geometrical imperfections, buckling

## Abstract

There are many possible sources of potential geometrical inaccuracies in each layer of corrugated board during its manufacture. These include, among others, the processes of wetting the corrugated layers during profiling, the process of accelerated drying, the gluing process, and any mechanical impact of the pressure rollers on the cardboard. Work taking into account all the above effects in numerical modeling is not well described in the literature. Therefore, this article presents a simple and practical procedure that allows us to easily account for geometric imperfections in the calculation of the effective stiffness of corrugated board. As a main tool, the numerical homogenization based on the finite element method (FE) was used here. In the proposed procedure, a 3D model of a representative volumetric element (RVE) of a corrugated board is first built. The numerical model can include all kinds of geometrical imperfections and is used to calculate the equivalent tensile and bending stiffnesses. These imperfections were included in the 3D numerical model by appropriate modeling of individual layers, taking into account their distorted shape, which was obtained on the basis of a priori buckling analysis. This paper analyzes different types of buckling in order to find the most representative one. The proposed procedure is easy to implement and fully scalable.

## 1. Introduction

Currently, the growing consumption and the increase in the number of individual shipments make it necessary to ensure the safe storage and transport of goods. At the same time, more emphasis is placed on ecology, which is why companies quickly began to abandon plastic, which not only harms the environment, but also has a negative impact on the reputation of enterprises. For these reasons, corrugated cardboard has begun to conquer the packaging market. It owes its huge popularity to numerous advantages that largely meet the needs of today. Cardboard packaging is recyclable, easy to dispose of, biodegradable, durable under appropriate conditions, and takes up little space before folding. In addition to the positives for the environment, there are also useful properties for companies. Corrugated cardboard products can be easily shaped by adding holes, ventilation holes, or by printing a brand logo on them. It is also possible to create boxes with perforations, which are often used in the food industry. Such packages are opened by tearing off a part of the material along the previously designed perforations, and then immediately put on the shelf. Such a procedure allows for significant time savings, which brings profits for large companies.

In addition to all the above-mentioned advantages, packaging should, above all, effectively protect the goods. For this reason, the load-bearing capacity of packaging has been studied by scientists since the 1950s. The first methods were analytical methods determined for boxes with a rectangular base. In 1952, Kellicutt and Landt introduced an approach based on box perimeter, overall ring crush strength, and paper and box constants [1]. Four years later, Maltenfort presented the box compressive strength depending on the critical force, paper parameters, box dimensions, and empirical constants [2]. In 1963, McKee et al. proposed a relationship that has been commonly used in the packaging industry for many years to estimate the compressive strength of cardboard boxes [3]. The popular McKee formula is based on the edge crush resistance (ECT) value, flexural stiffnesses, box perimeter, and correction factors. Due to the simplicity of the formula, it is possible to obtain a result quickly, but it can only be used for simple standard boxes. In the following years, scientists tried to extend and modify the McKee formula. In 1985, Allerby et al. changed the constants and exponents [4] and, in 1987, Schrampfer et al. extended the approach to new cardboard cutting methods and equipment [5]. In 1993, Batelka and Smith included the dimensions of the packaging in the formula [6].

In practice, the compressive strength of the packaging depends on many factors related to the construction and storage conditions [7]. From the construction point of view, attention should be paid to aspects, such as openings, ventilation holes, perforations, and offsets [8,9,10,11,12], because they significantly affect the behavior of the box under pressure. Load-bearing capacity is also influenced by moisture content [13,14], stacking load [15], storage time and conditions [16], and many other factors.

With the development of technology, numerical computations have become popular. The well-known finite element method (FEM) is often used to determine the compressive strength of packaging. Urbanik and Frank compared the compressive strength of boxes calculated using FEM with the results of the McKee formula extended by Poisson’s ratio [17]. Furthermore, FEM simulations were used by Nordstrand and Carlsson to compare analytical, experimental, and numerical values of transverse shear moduli [18,19]. The issue of transverse shear was studied by Aviles et al. [20] and Garbowski et al. [21,22], where the role of transversal shear stiffness in orthotropic sandwich material is presented. Urbanik and Saliklis used FEM to observe buckling and post-buckling phenomena in cardboard boxes [23]. Maneengam et al. used the FE model to study the vibration and damping characteristics of honeycomb sandwich structures reinforced with carbon nanotubes [24]. Corrugated board reinforced with metal liners has also been investigated by Gu et al., where uniaxial compression tests were conducted to analyze the damage and failure modes [25]. Sohrabpour and Hellström presented a review of analytical and numerical methods for estimating box compressive strength [26].

Due to the orthotropy of the paper and layered structure, corrugated board is quite difficult to analyze numerically. To facilitate the computations, the homogenization process is used, which consists of replacing a complicated cardboard cross-section with a homogeneous board with equivalent parameters. Homogenization methods can be divided into analytical and numerical. In analytical approaches, the equations of the classical theory of material strength and the classical theory of laminates are used [27]. Numerical homogenization is based on a finite element method framework. In this study, a method based on strain energy equivalence between a 3D cardboard model and a flat plate is used. This homogenization procedure was introduced by Biancolini [28] and later extended by Garbowski and Gajewski to include transversal shear stiffnesses [29]. In 2003, Hohe presented a representative element of heterogeneous and homogenized structures derived from the strain energy equations [30]. Homogenization was also used to determine substitute panel parameters, such as membrane and bending characteristics [31] and torsional stiffness [32]. In 2018, a multiscale asymptotic homogenization was used by Ramírez-Torres et al. for layered hierarchical structure analysis [33,34]. Suarez et al. used numerical homogenization to analyze seating made of multi-wall corrugated cardboard [35]. Garbowski et al. presented the influence of different creasing and perforation on cardboard parameters [36]. In 2022, Mrówczyński et al. presented non-local sensitivity analysis in the optimal design of three- [37] and five-layered [38] corrugated cardboard. A review of homogenization methods and their accuracy was made by Nguyen-Minh et al., based on analysis of corrugated panels [39].

During the production process, the corrugated board may be deformed due to changes in temperature and humidity. Two types of emerging imperfections can be distinguished, namely global and local. A model describing systematic, large-scale deviation from the intended flat shape of cardboard was presented by Beck and Fischerauer [40]. However, more attention was paid to local imperfections. In 1995, Nordstrand presented the effect of the size of imperfections on the compressive strength of cardboard boxes [41], and in 2004 extended the nonlinear buckling analysis of Rhodes and Harvey orthotropic plates to include local imperfections [42]. Lu et al. investigated the effect of imperfections on the mechanical properties of cardboard during compression [43]. The problem of non-ideal shape during bending of double-walled corrugated cardboard was analyzed analytically by Garbowski and Knitter-Piątkowska [44]. Mrówczyński et al. presented a method of including the initial imperfections in the analysis of single-walled cardboard [45]. Recently Cillie and Coetzee presented an experimental and numerical study on the in-plane compression of corrugated paperboard panels with global and local imperfections [46].

In the article, the authors focused on the issue of local imperfections in double-walled corrugated board. The method presented here allows us to quickly and easily obtain the effective cardboard stiffnesses reduction due to the imperfect shape of its component layers. The described approach is an extension of the homogenization method proposed in the authors’ previous works. Due to the specificity of the production process and very thin layers of paper, cardboard always has some imperfections, so it is important to be able to include this aspect in determining its mechanical parameters. The approach proposed in this paper extends the discussion carried out in our previous work [45], where the influence of imperfections on the stiffness of three-ply cardboard was investigated. The mechanics of both types differ significantly and, therefore, in this work other techniques, adequate to solve this problem, were used. The extension consists of taking into account the influence of the geometrical imperfections of individual layers on the calculated cross-section’s effective stiffness. These imperfections were taken into account in the model by appropriate modeling of geometrical imperfections, which were determined in a priori buckling analysis and were appropriately included using the innovative technique based on numerical homogenization of double-walled corrugated board. Thanks to this approach, it is possible not only to quickly obtain homogenized stiffnesses data for complex cross-sections, but also to easily take into account the effects of geometrical imperfections.

## 2. Materials and Methods

### 2.1. Corrugated Cardboard–Material and Geometry

**Figure 1:**
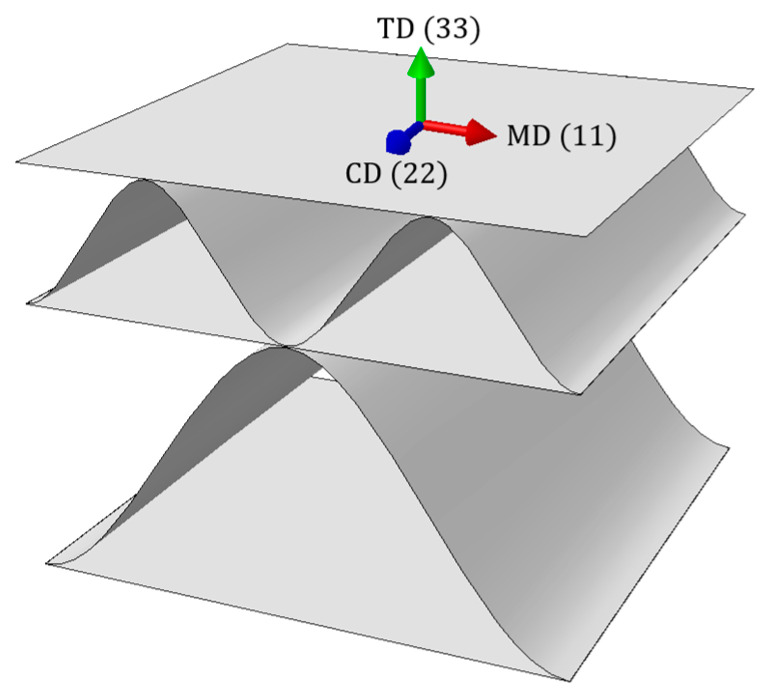
Corrugated cardboard orientation.

**Figure 2:**
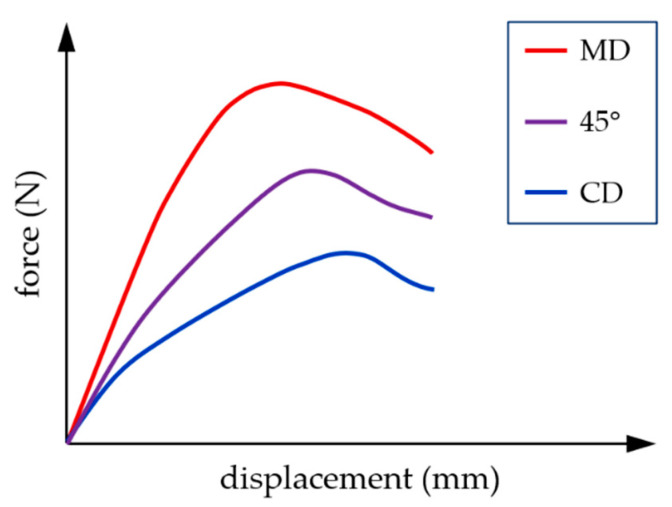
Mechanical behavior of cardboard in different material directions.

Corrugated cardboard is a composite material made of several flat and corrugated layers of paper, called “liners” and “flutings”, respectively. Due to the fibrous structure of the paper, the cardboard is characterized by a strong orthotropy. For this reason, the mechanical properties of the entire composite depend on the direction of fiber arrangement in individual layers. Two main directions can be distinguished, namely the machine direction (MD) and cross direction (CD) (see Figure 1). They result from the production process, in which layers of paper are rolled from multi-tone bales. The cardboard in the MD is stiffer but less ductile than in the CD (see Figure 2). The lower strength of the layers in the cross direction is partly compensated by the corrugation of the layers, which “adds” the material in this direction.

Linear elastic orthotropic material can be represented by the stress–strain relationship, as follows:

(1) $$\left[\begin{array}{c} \varepsilon_{11}\\ \varepsilon_{22}\\ 2\varepsilon_{12}\\ 2\varepsilon_{13}\\ 2\varepsilon_{23} \end{array}\right]=\left[\begin{array}{ccccc} 1/E_{1} & -\nu_{21}/E_{2} & 0 & 0 & 0\\ -\nu_{12}/E_{1} & 1/E_{2} & 0 & 0 & 0\\ 0 & 0 & 1/G_{12} & 0 & 0\\ 0 & 0 & 0 & 1/G_{13} & 0\\ 0 & 0 & 0 & 0 & 1/G_{23} \end{array}\right]\left[\begin{array}{c} \sigma_{11}\\ \sigma_{22}\\ \sigma_{12}\\ \sigma_{13}\\ \sigma_{23} \end{array}\right],$$

where $\varepsilon_{ij}$ and $\sigma_{ij}$ are the strain and stress vector components, $E_{1}$ and $E_{2}$ are the Young’s moduli in the machine and cross directions, respectively, $\nu_{12}$ and $\nu_{21}$ are the Poisson’s coefficients, $G_{12}$ is the in-plane shear modulus, $G_{13}$ and $G_{23}$ are the transverse shear moduli. The compliance/stiffness matrix is symmetrical, so the relationship between Poisson’s ratios can be written as follows:

(2) $$\frac{\nu_{12}}{E_{1}}=\frac{\nu_{21}}{E_{2}}.$$

In this work, the paper layers were modeled using the linear elastic classical orthotropy described above. The material data were taken from the literature [28] and are presented in Table 1. The thickness of both flat and corrugated layers was assumed to be 0.30 mm.

### 2.2. Imperfections–Numerical Study

**Figure 3:**
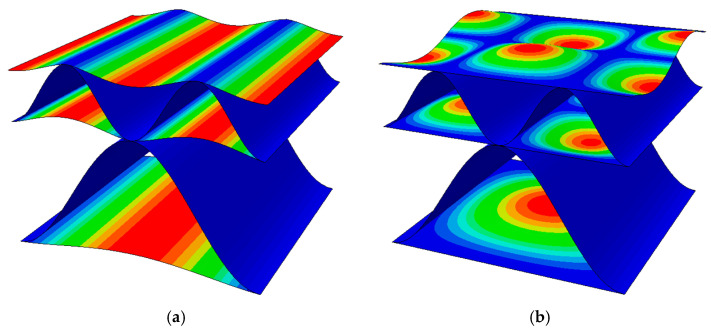
Imperfection shapes of double-walled corrugated board corresponding to (**a**) compression in the MD; (**b**) compression in the CD.

The main goal of the work is the numerical analysis of many cases of a corrugated board samples with imperfections and their impact on the stiffness values. In Figure 3, two imperfection shapes are presented; these were considered in this study. The first shape is the most common and corresponds to compression in the machine direction (see Figure 3a). It is formed as a result of thermal and moisture processes during the production of cardboard. The second imperfection mode (see Figure 3b) corresponds to the compression of the sample in the cross direction. This buckling shape was selected in [45] as the most representative for single-walled corrugated board. In both cases, it was assumed that only liners are involved in buckling analysis, and flutes (waves) only support the liners in the right position.

**Figure 4:**
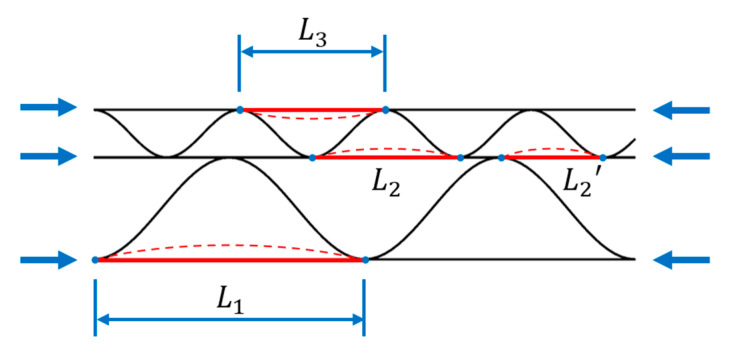
Buckling lengths of corrugated board liners.

All calculations were made for double-walled cardboard composed of high B flute and low E flute (EB cardboard). In Table 2, the geometry of the corrugated layers is presented. The variants also include four imperfection levels, namely 0, 1, 2, and 3% for both imperfection shapes. The amount of the imperfection was taken as a certain part of the buckling length of the liners. Three different buckling lengths can be specified for the analyzed double-walled corrugated board (see Figure 4). The lengths $L_{1}$ and $L_{3}$ correspond to the periods of the B and E waves, respectively. The buckling length ${L_{2}}^{\prime }$ can take different values depending on the relative position of the crests of E and B waves. The maximum possible value of $L_{2}$ is equal to the period of the lower wave, but most often every second segment is divided into two parts by the top of the B wave. For this reason, the buckling length of the middle liner was assumed to be 2/3 of the E wave period.

**Figure 5:**
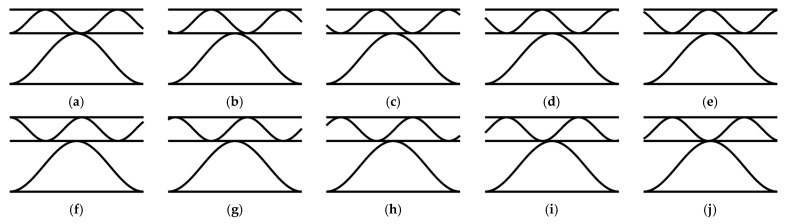
Cross-sections of corrugated board along the wave with offsets of (**a**) 0%, (**b**) 10%, (**c**) 20%, (**d**) 30%, (**e**) 40%, (**f**) 50%, (**g**) 60%, (**h**) 70%, (**i**) 80%, and (**j**) 90%.

Additionally, due to the irregular arrangement of the B and E flutes, the effect of the flute offset on the reduction in cardboard stiffness was verified. For this purpose, for each set of shape and level of imperfection, 10 cases of shifting the lower wave relative to the higher one, ranging from 0% to 90% in increments of 10%, were considered. In Figure 5, all simulated cross-sections are shown.

Taking into account all the variants described above, each case can be marked with the symbol XX-Y-ZZ. The first two letters XX can be “MD” or “CD”, which indicates the imperfection shape corresponding to compression in the MD and CD, respectively. The third sign Y represents the imperfection value and can be 0, 1, 2, or 3, and ZZ is the offset of the lower wave relative to the higher flute and can be 00, 10, 20, 30, 40, 50, 60, 70, 80, or 90.

### 2.3. Homogenization Procedure

The effect of imperfections on the effective stiffnesses of corrugated board was investigated with the numerical homogenization method proposed by Biancolini in 2005 and extended by Garbowski and Gajewski in 2021. It consists of ensuring the equivalence of the strain energy between the full 3D model of a representative volume element (RVE) and a simplified model of a flat 2D plate. The RVE is a small repeatable fragment of the full 3D model, which in this case is one period of the higher flute of the corrugated board. In the paper, only the main assumptions of the homogenization method used were presented. Detailed information and full derivation can be found in [29].

**Figure 6:**
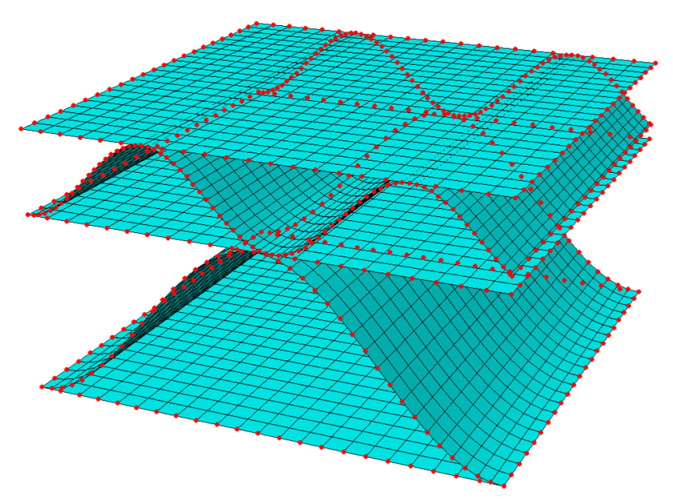
Finite element mesh and external nodes (in red color).

The homogenization procedure is based on the finite element method. Displacements from linear analysis can be represented by the following basic formula:

(3) $$K_{e}\;u_{e}=F_{e},$$

where $K_{e}$ is the stiffness matrix of the RVE after static condensation, $u_{e}$ is a displacement vector of the external nodes, and $F_{e}$ is the vector of external forces applied to the considered nodes. The finite element mesh and external nodes are presented in Figure 6.

The static condensation procedure is based on the removal of unnecessary unknown degrees of freedom (DOF) and leaving the considered degrees of freedom (called principal DOFs or primary unknowns). In the case of cardboard, the primary unknowns are external RVE nodes. This results in bringing the stiffness matrix only to the external nodes of the model. The FE stiffness matrix after static condensation can be calculated from the following equation:

(4) $$K_{e}=K_{ee}-K_{ei}\;K_{ii}^{-1}K_{ie},$$

where the components of the stiffness matrix are written for internal (subscript i) and external (subscript e) nodes, as follows:

(5) $$\left[\begin{array}{cc} K_{ee} & K_{ei}\\ K_{ie} & K_{ii} \end{array}\right]\left[\begin{array}{c} u_{e}\\ u_{i}\; \end{array}\right]=\left[\begin{array}{c} F_{e}\\ 0 \end{array}\right].$$

By the static condensation, the equation of the total elastic strain energy is simplified to the multiplication of the nodal displacements and the external forces acting on these nodes, as follows:

(6) $$E=\frac{1}{2}u_{e}^{T}\;F_{e}.$$

Maintaining the appropriate properties of the simplified model is ensured by the balance of the total energy between the 3D model of corrugated board and the 2D plate model. The appropriate definition of displacements at the external edges of the RVE and allowing for bending and membrane behaviors works to ensure the energy balance. The relationship between generalized displacements and strains can be represented by the transformation matrix $H_{j}$, as follows:

(7) $$u_{j}=H_{j}\;\epsilon_{j}.$$

and considering a single node ($x_{j}=x$, $y_{j}=y$, $z_{j}=z$) the above equation can be written in matrix form, as follows:

(8) $${\left[\begin{array}{c} u_{x}\\ u_{y}\\ u_{z}\\ \theta_{x}\\ \theta_{y} \end{array}\right]}_{j}={\left[\begin{array}{cccccccc} x & 0 & y/2 & xz & 0 & yz/2 & z/2 & 0\\ 0 & y & x/2 & 0 & yz & xz/2 & 0 & z/2\\ 0 & 0 & 0 & -x^{2}/2 & -y^{2}/2 & -xy/2 & x/2 & y/2\\ 0 & 0 & 0 & 0 & -y & -x/2 & 0 & 0\\ 0 & 0 & 0 & x & 0 & y/2 & 0 & 0 \end{array}\right]}_{j}\;{\left[\begin{array}{c} \varepsilon_{x}\\ \varepsilon_{y}\\ \gamma_{xy}\\ \kappa_{x}\\ \kappa_{y}\\ \kappa_{xy}\\ \gamma_{xz}\\ \gamma_{yz} \end{array}\right]}_{j}.$$

Substituting the basic FEM equation and the relationship between generalized displacements and strains into Equation (6) results in the following:

(9) $$E=\frac{1}{2}u_{e}^{T}\;K\;u_{e}=\frac{1}{2}\epsilon_{e}^{T}\;H_{e}^{T}\;K\;H_{e}\;\epsilon_{e}$$

and considering a finite element subjected to standard load conditions, such as bending, stretching, shear, elastic internal energy, this can be represented by the following equation:

(10) $$E=\frac{1}{2}\epsilon_{e}^{T}\;H_{k}\;\epsilon_{e}\left\{area\right\},$$

where the matrix $H_{k}$ is the RVE stiffness matrix, which can be determined from the following:

(11) $$H_{k}=\frac{H_{e}^{T}\;K\;H_{e}}{area}.$$

The stiffness matrix $H_{k}$ consists of several submatrices corresponding to the basic load conditions, as follows:

(12) $$H_{k}=\left[\begin{array}{ccc} A_{3\times 3} & B_{3\times 3} & \\ B_{3\times 3} & D_{3\times 3} & \\ & & R_{2\times 2} \end{array}\right],$$

where A represents the tensile and in-plane shear stiffnesses, D represents the bending and torsional stiffnesses, B is the coupling subarray of the tension and bending stiffnesses, and R is the transversal shear stiffnesses.

The stiffness values in subarray A do not depend on the position of a neutral axis. This is not true in the case of subarray B, in which the values change depending on the position of the neutral axis. In most symmetrical cases, the stiffness matrix B is a zero matrix, unlike for asymmetric cross-sections (e.g., multi-walled corrugated cardboard), for which some components of the matrix B are not zero, which affects the stiffness in the subarray D. This effect can be suppressed in two ways. The first is to choose the position of the neutral axis so as to minimize the values in the subarray B. An alternative solution is to calculate the uncoupled matrix D from the following formula:

(13) $$D=D^{\prime }-BA^{-1}B,$$

where $D^{\prime }$ contains the original (coupled) bending and torsional stiffnesses for the non-zero matrix B.

Numerical computations consisted in applying the selected buckling shape and imperfection value, and then calculating the stiffness matrix of the RVE. In all analyses, the four-node quadrilaterals shell elements with full integration (S4 elements) were used [47]. All models were built in FE commercial software, from where the stiffnesses in all nodes were generated. Next, the effective stiffness matrix was determined using the homogenization method described above. An approximate global size equal to 0.1625 mm was assumed. Due to the different variants of offset, the number of elements changed. For the MD-0-00 case, the model consisted of 9922 nodes, 9760 elements, and 59,532 degrees of freedom. The sensitivity study of the mesh size was already studied in our previous works; therefore, here this part of the research is omitted. It is worth noting that in the presented homogenization method, the use of the finite element method is limited only to building a global stiffness matrix, and no formal loads or boundary conditions are defined. They are directly implemented through the $H_{e}$ matrix in Equation (11).

## 3. Results

The first step was to properly create the corrugated cardboard RVE model in FE software. In the cases of samples with imperfections, the geometry of the liners was changed and replaced with the correct buckling shape (see Figure 3). Then, the material parameters contained in Table 1 were assigned to both liners and waves. Following the homogenization procedure described in Section 2.3, stiffness matrices were determined for all analyzed cases. In Table 3, an example of the stiffness matrix $H_{k}$ is presented for a model without imperfections and with 0% offset. No buckling mode is assigned to this case, because the imperfection value is equal to zero; therefore, it was marked with the XX-0-00 symbol.

**Figure 7:**
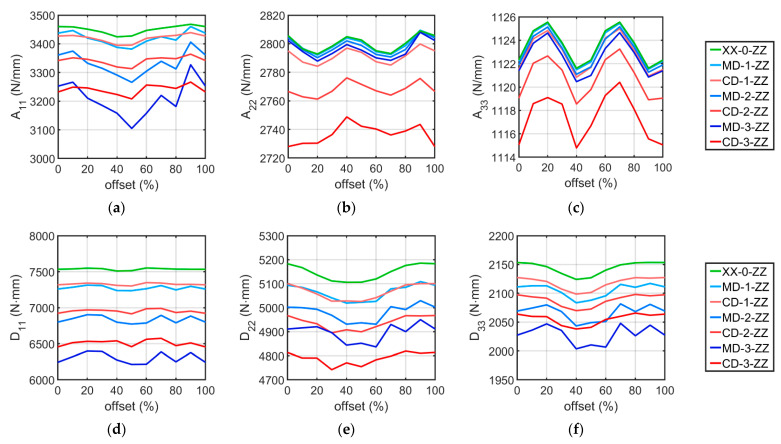
Influence of imperfections and offset on the cardboard stiffness of (**a**) $A_{11}$, (**b**) $A_{22}$, (**c**) $A_{33}$, (**d**) $D_{11}$, (**e**) $D_{22}$, and (**f**) $D_{33}$.

As expected, non-zero components of the B matrix appeared in the stiffness matrix $H_{k}$, which results from the asymmetry of the double-walled corrugated board sample. Table 4, Table 5, Table 6, Table 7, Table 8, Table 9 and Table 10 show the main diagonal components of the stiffness matrix for all analyzed cases. The components ${\left(*\right)}_{12}$ are omitted, but this does not create an error in the analysis because these elements are related to the components ${\left(*\right)}_{11}$ and ${\left(*\right)}_{12}$ in each stiffness matrix. The following tables do not list the elements of matrix B, but its effect on matrix D has been taken into account by applying Equation (13). The components of the R matrix were also omitted. The data contained in Table 4, Table 5, Table 6, Table 7, Table 8, Table 9 and Table 10 are also presented in the form of graphs in Figure 7.

**Figure 8:**
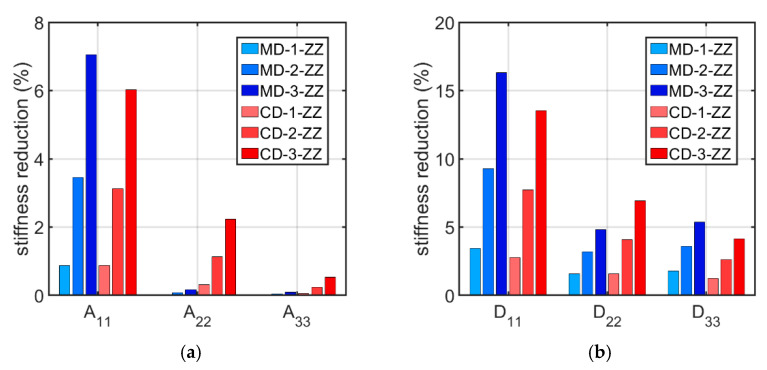
Average percentage stiffness reduction in (**a**) tensile stiffnesses and (**b**) bending stiffnesses.

For each shape and level of imperfection, the average stiffness values from all offset cases were calculated. Then, the obtained values for models with imperfections were compared to the average stiffnesses of perfectly shaped cardboard. In Figure 8, the tensile and bending stiffness reduction due to geometrical imperfections is shown.

As can be seen in Table 4, Table 5, Table 6, Table 7, Table 8, Table 9 and Table 10 and Figure 7, the stiffnesses vary depending on the offset. Considering each set of shape and level of imperfection, the difference between the maximum and minimum stiffness values was calculated. Then, the obtained difference was related to the average stiffness obtained from all 10 offset cases, obtaining a percentage relative spread. In Table 11, the relative spread for all analyzed sets of imperfection is presented.

## 4. Discussion

All crucial results have been shown in Table 3, Table 4, Table 5, Table 6, Table 7, Table 8, Table 9, Table 10 and Table 11 and Figure 7 and Figure 8. In Table 3, an example of a stiffness matrix determined for the XX-0-00 model is presented, in which the submatrices A, B, D, and R are distinguished. The B matrix contains non-zero components because the XX-0-00 case cross-section, as in all other models, is asymmetric. For this reason, Equation (13) was used in all corrugated board cases to calculate the uncoupled matrix D, whose values are gathered in Table 4, Table 5, Table 6, Table 7, Table 8, Table 9 and Table 10. Comparing the stiffness values $D_{11}$, $D_{22}$, and $D_{33}$ from Table 3 and Table 4, it can be seen that the values in Table 4 are smaller, which results from the uncoupling of the D matrix.

Considering Table 4, Table 5, Table 6, Table 7, Table 8, Table 9 and Table 10 and Figure 7, where the stiffness results for all models are presented, it can be seen that as the level of imperfection increases, the cardboard stiffness decreases, as expected. The line diagrams also show that depending on the applied wave offset, the stiffnesses change their value. This is shown in more detail in Table 11, which shows the relative spread of the extreme stiffnesses. The greatest fluctuations occur for the $A_{11}$ stiffnesses, are slightly smaller for all bending stiffnesses, and are negligibly small for the $A_{22}$ and $A_{33}$ stiffnesses. In most cases, it is clear that the relative spread increases as the amount of imperfection increases. In addition, the data in this table show that the stiffnesses of the models with the MD imperfection shape are more sensitive to offset changes than the cases with the CD buckling mode.

Based on Table 4, Table 5, Table 6, Table 7, Table 8, Table 9 and Table 10 and Figure 7 and Figure 8, it can be concluded that geometric imperfections have a huge influence on the bending stiffness $D_{11}$. Large decreases were also noted for the stiffnesses $A_{11}$, $D_{22}$, and $D_{33}$. The effect of imperfections on the stiffness $A_{22}$ is small, and the effect is negligible on the $A_{33}$ stiffness. These results are very similar to those obtained from the analysis of the effect of imperfections on the stiffness of single-walled corrugated board conducted by Mrówczyński et al. [45], where the largest decreases were also obtained for $D_{11}$, and the smallest were obtained for $A_{33}$. The implementation of the MD buckling mode results in a greater average reduction in stiffnesses $A_{11}$, $D_{11}$, and $D_{33}$ compared to the CD imperfection shape, for which a higher average reduction occur for the stiffnesses of $A_{22}$, $A_{33}$, and $D_{22}$. These differences are not that significant and amount to a maximum of 2.75 % (for the stiffness $D_{11}$ between the MD-3-ZZ and CD-3-ZZ models).

To simplify computations and save computational time, it is desirable to select one case that should be computed as representative instead of carrying out an entire numerical study. To determine this, several observations from the conducted analyses should be considered. The imperfection shape of the compression in the CD has smaller relative spread values than the buckling mode of compression in the MD, so the change in the offset does not affect the stiffnesses so significantly. In addition, considering the MD shape does not significantly reduce the stiffnesses $A_{22}$ and $A_{33}$, which seems to be an unfavorable effect. Based on the calculations, it can be concluded that a reasonable solution is to choose the CD imperfection shape as a representative model. Due to the quite small relative spread, the choice of a representative offset value is not so important, so its value can be chosen arbitrarily. The conclusions drawn from the presented numerical study are consistent with the work of Mrówczyński et al. [45], in which the authors showed that the CD imperfection shape is also the most representative buckling mode for single-walled corrugated cardboard.

All the observations discussed above prove that there is a direct influence of imperfections on the stiffnesses **A**, **B**, and **D**. However, the basic question arises whether it is possible to verify the presented theoretical approach with experimental data. Although testing corrugated board is not very laborious or particularly expensive, nevertheless validating the theoretical analysis presented here is not an easy task, because, firstly, it is practically impossible to produce corrugated board with a given level of imperfection and, secondly, it is also very laborious to check the actual level of imperfection in the cardboard produced. Nevertheless, below, we present a comparison (validation) of the results obtained using the method presented here and the method presented in paper [44] in comparison with the results of laboratory tests conducted by Czechowski et al. and presented in paper [48].

All experimental details are described in [48], while analytical assumptions, in which imperfections are also included, are given in [44]. Here, only the results of the experimental campaign (namely, results of 4-point bending tests of various asymmetric double-walled boards) are shown together with the results obtained using numerical and analytical models. All these results are presented in Table 12, together with the results obtained using the methods presented here with the same amount of imperfection of flat layers as assumed in [44]. It is worth noting that only one of all stiffnesses is checked, namely the bending stiffness in MD. The obtained results do not differ by more than 5% from the results obtained using the analytical model, while the average absolute error (compared to the experimental data) was 7.5%.

## 5. Conclusions

The article presents an approach of numerical homogenization of double-walled corrugated board, taking into account the initial imperfections of its layers. The creation of many cardboard models in various configurations made it possible to study various aspects of the considered problem. In the study, corrugated cardboard was homogenized using the numerical method based on the strain energy equivalence between a representative 3D model and a simplified plate. The analysis of five-layered cardboard with imperfections is not such an easy task. The cross-sectional shape of the cardboard depends on the storage and load conditions. For this reason, it is important to indicate a representative case that adequately reflects the properties of the material.

Based on the conducted analyses, it can be seen that the bending stiffnesses and the tensile stiffness along the wave are the most sensitive to the imperfect shape of the cardboard. Calculations were made for a wide range of cases, which allowed us to choose a representative imperfection model. The conclusions drawn from the numerical analyses are consistent with the research that was carried out in the previous work for single-walled corrugated board.

## Figures and Tables

**Table 1:** Material data for orthotropic constitutive model of corrugated cardboard layers.

Layers	$E_{1}$	$E_{2}$	$\nu_{12}$	$G_{12}$	$G_{13}$	$G_{23}$
	(MPa)	(MPa)	(-)	(MPa)	(MPa)	(MPa)
Liners	3326	1694	0.34	859	429.5	429.5
Fluting	2614	1532	0.32	724	362	362

**Table 2:** Geometric parameters of cardboard flutes.

Wave (Flute)	Wavelength (mm)	Height (mm)	Take-up Factor (-)
B	6.5	2.46	1.32
E	3.5	1.15	1.27

**Table 3:** Constitutive stiffness matrix $H_{k}$ for the XX-0-00 model.

		A and B			B and D			R	
		1	2	3	1	2	3	4	5
**A and B**	1	3460	630	0	741	128	0		
	2	630	2806	0	128	732	0		
	3	0	0	1122	0	0	296		
**B and D**	1	741	128	0	7692	1357	0		
	2	128	732	0	1357	5375	0		
	3	0	0	296	0	0	2231		
**R**	4							206	0
	5							0	167

**Table 4:** The selected stiffnesses in models without imperfections and with different offsets.

	XX-0-00	XX-0-10	XX-0-20	XX-0-30	XX-0-40	XX-0-50	XX-0-60	XX-0-70	XX-0-80	XX-0-90
$A_{11}$ (N/mm)	3460	3459	3451	3441	3424	3427	3447	3454	3461	3468
$A_{22}$ (N/mm)	2806	2796	2793	2798	2805	2803	2795	2793	2800	2809
$A_{33}$ (N/mm)	1122	1125	1126	1124	1122	1122	1125	1126	1124	1122
$D_{11}$ (N⋅mm)	7533	7538	7549	7543	7510	7514	7552	7544	7536	7533
$D_{22}$ (N⋅mm)	5183	5167	5137	5111	5106	5106	5120	5151	5176	5186
$D_{33}$ (N⋅mm)	2153	2152	2146	2134	2124	2127	2140	2149	2153	2154

**Table 5:** The selected stiffnesses with 1% imperfections of compression in the MD shape and with different offsets.

	MD-1-00	MD-1-10	MD-1-20	MD-1-30	MD-1-40	MD-1-50	MD-1-60	MD-1-70	MD-1-80	MD-1-90
$A_{11}$ (N/mm)	3437	3446	3420	3408	3388	3382	3409	3425	3413	3460
$A_{22}$ (N/mm)	2806	2797	2792	2798	2804	2801	2794	2793	2799	2809
$A_{33}$ (N/mm)	1122	1125	1125	1124	1121	1122	1125	1125	1124	1122
$D_{11}$ (N⋅mm)	7259	7283	7314	7307	7238	7237	7263	7307	7247	7297
$D_{22}$ (N⋅mm)	5094	5085	5066	5041	5019	5023	5026	5079	5085	5108
$D_{33}$ (N⋅mm)	2111	2113	2113	2101	2084	2088	2096	2115	2110	2117

**Table 6:** The selected stiffnesses with 2% imperfections of compression in the MD shape and with different offsets.

	MD-2-00	MD-2-10	MD-2-20	MD-2-30	MD-2-40	MD-2-50	MD-2-60	MD-2-70	MD-2-80	MD-2-90
$A_{11}$ (N/mm)	3361	3375	3332	3314	3291	3266	3305	3339	3313	3406
$A_{22}$ (N/mm)	2804	2796	2790	2796	2802	2799	2793	2791	2796	2809
$A_{33}$ (N/mm)	1122	1124	1125	1123	1121	1122	1124	1125	1123	1121
$D_{11}$ (N⋅mm)	6799	6848	6902	6896	6798	6772	6787	6893	6790	6884
$D_{22}$ (N⋅mm)	5002	5000	4993	4968	4931	4937	4931	5004	4992	5029
$D_{33}$ (N⋅mm)	2069	2074	2080	2068	2043	2049	2051	2082	2068	2081

**Table 7:** The selected stiffnesses with 3% imperfections of compression in the MD shape and with different offsets.

	MD-3-00	MD-3-10	MD-3-20	MD-3-30	MD-3-40	MD-3-50	MD-3-60	MD-3-70	MD-3-80	MD-3-90
$A_{11}$ (N/mm)	3252	3266	3210	3185	3158	3105	3158	3220	3181	3327
$A_{22}$ (N/mm)	2802	2794	2788	2793	2799	2796	2790	2788	2793	2808
$A_{33}$ (N/mm)	1121	1124	1125	1123	1120	1121	1123	1125	1123	1121
$D_{11}$ (N⋅mm)	6243	6320	6399	6393	6276	6213	6216	6387	6250	6377
$D_{22}$ (N⋅mm)	4911	4916	4921	4896	4844	4852	4837	4930	4900	4950
$D_{33}$ (N⋅mm)	2027	2036	2047	2036	2004	2010	2007	2048	2026	2045

**Table 8:** The selected stiffnesses with 1% imperfections of compression in the CD shape and with different offsets.

	CD-1-00	CD-1-10	CD-1-20	CD-1-30	CD-1-40	CD-1-50	CD-1-60	CD-1-70	CD-1-80	CD-1-90
$A_{11}$ (N/mm)	3427	3429	3423	3411	3395	3395	3419	3426	3429	3439
$A_{22}$ (N/mm)	2795	2787	2784	2790	2797	2794	2787	2785	2792	2800
$A_{33}$ (N/mm)	1121	1124	1125	1123	1121	1122	1124	1125	1123	1121
$D_{11}$ (N⋅mm)	7318	7329	7342	7335	7310	7302	7348	7342	7323	7324
$D_{22}$ (N⋅mm)	5100	5082	5057	5027	5028	5026	5042	5069	5095	5100
$D_{33}$ (N⋅mm)	2127	2125	2121	2107	2099	2101	2115	2122	2127	2126

**Table 9:** The selected stiffnesses with 2% imperfections of compression in the CD shape and with different offsets.

	CD-2-00	CD-2-10	CD-2-20	CD-2-30	CD-2-40	CD-2-50	CD-2-60	CD-2-70	CD-2-80	CD-2-90
$A_{11}$ (N/mm)	3342	3351	3346	3335	3319	3313	3347	3351	3348	3364
$A_{22}$ (N/mm)	2767	2763	2761	2767	2776	2772	2767	2764	2769	2776
$A_{33}$ (N/mm)	1119	1122	1123	1121	1119	1120	1122	1123	1121	1119
$D_{11}$ (N⋅mm)	6922	6956	6969	6965	6954	6914	6985	6990	6932	6952
$D_{22}$ (N⋅mm)	4967	4947	4933	4895	4908	4900	4921	4943	4967	4966
$D_{33}$ (N⋅mm)	2097	2094	2092	2077	2070	2073	2086	2093	2098	2096

**Table 10:** The selected stiffnesses with 3% imperfections of compression in the CD shape and with different offsets.

	CD-3-00	CD-3-10	CD-3-20	CD-3-30	CD-3-40	CD-3-50	CD-3-60	CD-3-70	CD-3-80	CD-3-90
$A_{11}$ (N/mm)	3232	3249	3246	3234	3223	3208	3256	3253	3244	3267
$A_{22}$ (N/mm)	2728	2730	2730	2736	2749	2742	2740	2736	2739	2744
$A_{33}$ (N/mm)	1115	1119	1119	1119	1115	1117	1119	1120	1118	1116
$D_{11}$ (N⋅mm)	6455	6514	6532	6528	6540	6458	6561	6574	6473	6511
$D_{22}$ (N⋅mm)	4814	4791	4790	4742	4771	4755	4783	4799	4819	4810
$D_{33}$ (N⋅mm)	2064	2060	2059	2044	2038	2041	2055	2060	2066	2062

**Table 11:** Relative spread of the extreme stiffnesses for the selected shape and level of imperfection.

Case	Relative Spread (%)					
	$A_{11}$	$A_{22}$	$A_{33}$	$D_{11}$	$D_{22}$	$D_{33}$
MD-3-ZZ	6.9	0.7	0.4	2.9	2.3	2.2
MD-2-ZZ	4.2	0.7	0.4	1.9	2.0	1.9
MD-1-ZZ	2.3	0.6	0.4	1.1	1.8	1.6
XX-0-ZZ	1.3	0.6	0.4	0.6	1.6	1.4
CD-1-ZZ	1.3	0.6	0.4	0.6	1.5	1.4
CD-2-ZZ	1.5	0.5	0.4	1.1	1.5	1.3
CD-3-ZZ	1.8	0.8	0.5	1.8	1.6	1.3

**Table 12:** Bending stiffness in MD for all considered models.

Board ID	Face-Up	EXP (Mean) [48]	FEM [48]	Analytical [44]	Present Model
		(Nm)	(Nm)	(Nm)	(Nm)
Board 1	EB	8.32	7.62	7.13	7.54
	BE	8.47	7.58	7.84	8.34
Board 2	EB	10.97	9.88	11.15	11.78
	BE	11.58	9.81	11.65	12.20
Board 3	EB	7.25	7.61	7.15	7.54
	BE	9.50	7.53	7.85	8.16
Board 4	EB	9.10	7.53	7.24	7.69
	BE	11.10	7.45	7.98	8.26
Board 5	EB	11.46	10.42	10.89	11.33
	BE	12.97	10.37	11.52	12.02
Board 6	EB	8.20	8.45	8.86	9.01
	BE	9.12	8.40	9.27	9.88

## Data Availability

The data presented in this study are available on request from the corresponding author.

## References

[1] Kellicutt K., Landt E. (1952). Development of design data for corrugated fiberboard shipping containers. TAPPI J..

[2] Maltenfort G. (1956). Compression strength of corrugated containers. Fibre Contain..

[3] McKee R.C., Gander J.W., Wachuta J.R. (1963). Compression strength formula for corrugated boxes. Paperboard Packag..

[4] Allerby I.M., Laing G.N., Cardwell R.D. (1985). Compressive strength—From components to corrugated containers. Appita Conf. Notes.

[5] Schrampfer K.E., Whitsitt W.J., Baum G.A. (1987). Combined Board Edge Crush (ECT) Technology.

[6] Batelka J.J., Smith C.N. (1993). Package Compression Model.

[7] Frank B. (2014). Corrugated Box Compression—A Literature Survey. Packag. Technol. Sci..

[8] Garbowski T., Gajewski T., Grabski J.K. (2021). Estimation of the compressive strength of corrugated cardboard boxes with various openings. Energies.

[9] Archaviboonyobul T., Chaveesuk R., Singh J., Jinkarn T. (2020). An analysis of the influence of hand hole and ventilation hole design on compressive strength of corrugated fiberboard boxes by an artificial neural network model. Packag. Technol. Sci..

[10] Fadiji T., Coetzee C.J., Opara U.L. (2016). Compression strength of ventilated corrugated paperboard packages: Numerical modelling, experimental validation and effects of vent geometric design. Biosyst. Eng..

[11] Garbowski T., Gajewski T., Grabski J.K. (2021). Estimation of the compressive strength of corrugated cardboard boxes with various perforations. Energies.

[12] Mrówczyński D., Garbowski T., Knitter-Piątkowska A. (2021). Estimation of the Compressive Strength of Corrugated Board Boxes with Shifted Creases on the Flaps. Materials.

[13] Stott R.A. (2017). Compression and stacking strength of corrugated fibreboard containers. Appita J..

[14] Junli W., Quancheng Z. (2006). Effect of moisture content of corrugated box on mechanical properties. J. Lanzhou Jiaotong Univ..

[15] Gallo J., Cortés F., Alberdi E., Goti A. (2021). Mechanical behavior modeling of containers and octabins made of corrugated cardboard subjected to vertical stacking loads. Materials.

[16] Zhang Y.-L., Chen J., Wu Y., Sun J. (2011). Analysis of hazard factors of the use of corrugated carton in packaging low-temperature yogurt during logistics. Procedia Environ. Sci..

[17] Urbanik T.J., Frank B. (2006). Box compression analysis of world-wide data spanning 46 years. Wood Fiber Sci..

[18] Nordstrand T., Carlsson L. (1997). Evaluation of transverse shear stiffness of structural core sandwich plates. Compos. Struct..

[19] Nordstrand T. (2003). Basic Testing and Strength Design of Corrugated Board and Containers. Ph.D. Thesis.

[20] Avilés F., Carlsson L.A., May-Pat A. (2012). A shear-corrected formulation of the sandwich twist specimen. Exp. Mech..

[21] Garbowski T., Gajewski T., Grabski J.K. (2020). Torsional and transversal stiffness of orthotropic sandwich panels. Materials.

[22] Garbowski T., Gajewski T., Grabski J.K. (2020). Role of transverse shear modulus in the performance of corrugated materials. Materials.

[23] Urbanik T.J., Saliklis E.P. (2003). Finite element corroboration of buckling phenomena observed in corrugated boxes. Wood Fiber Sci..

[24] Maneengam A., Siddique M.J., Selvaraj R., Kakaravada I., Arumugam A.B., Singh L.K., Kumar N. (2022). Influence of multi-walled carbon nanotubes reinforced honeycomb core on vibration and damping responses of carbon fiber composite sandwich shell structures. Polym. Compos..

[25] Gu F., Chen L., Zhu X., Lu X., Fang D. (2022). Fabrication and uniaxial compression mechanical behavior of composite corrugated-core sandwich cylinder with thin-wall metal liner. Mech. Adv. Mater. Struct..

[26] Sohrabpour V., Hellström D. Models and software for corrugated board and box design. Proceedings of the 18th International Conference on Engineering Design (ICED 11).

[27] Allaoui S., Benzeggagh M.L., Aboura Z., Talbi N. (2004). Elastic behaviour of corrugated cardboard: Experiments and modeling. Compos. Struct..

[28] Biancolini M.E. (2005). Evaluation of equivalent stiffness properties of corrugated board. Compos. Struct..

[29] Garbowski T., Gajewski T. (2021). Determination of transverse shear stiffness of sandwich panels with a corrugated core by numerical homogenization. Materials.

[30] Hohe J. (2003). A direct homogenization approach for determination of the stiffness matrix for microheterogeneous plates with application to sandwich panels. Compos. Part B.

[31] Buannic N., Cartraud P., Quesnel T. (2003). Homogenization of corrugated core sandwich panels. Compos. Struct..

[32] Abbès B., Guo Y.Q. (2010). Analytic homogenization for torsion of orthotropic sandwich plates. Appl. Compos. Struct..

[33] Ramírez-Torres A., Penta R., Rodríguez-Ramos R., Merodio J., Sabina F.J., Bravo-Castillero J., Guinovart-Díaz R., Preziosi L., Grillo A. (2018). Three scales asymptotic homogenization and its application to layered hierarchical hard tissues. Int. J. Solids Struct..

[34] Ramírez-Torres A., Di Stefano S., Grillo A., Rodríguez-Ramos R., Merodio J., Penta R. (2018). An asymptotic homogenization approach to the microstructural evolution of heterogeneous media. Int. J. Non Linear Mech..

[35] Suarez B., Muneta M.L.M., Sanz-Bobi J.D., Romero G. (2021). Application of homogenization approaches to the numerical analysis of seating made of multi-wall corrugated cardboard. Compos. Struct..

[36] Garbowski T., Knitter-Piątkowska A., Mrówczyński D. (2021). Numerical homogenization of multi-layered corrugated cardboard with creasing or perforation. Materials.

[37] Mrówczyński D., Knitter-Piątkowska A., Garbowski T. (2022). Non-Local Sensitivity Analysis and Numerical Homogenization in Optimal Design of Single-Wall Corrugated Board Packaging. Materials.

[38] Mrówczyński D., Knitter-Piątkowska A., Garbowski T. (2022). Optimal Design of Double-Walled Corrugated Board Packaging. Materials.

[39] Nguyen-Minh N., Tran-Van N., Bui-Xuan T., Nguyen-Thoi T. (2019). Static analysis of corrugated panels using homogenization models and a cell-based smoothed mindlin plate element (CS-MIN3). Front. Struct. Civ. Eng..

[40] Beck M., Fischerauer G. (2022). Modeling Warp in Corrugated Cardboard Based on Homogenization Techniques for In-Process Measurement Applications. Appl. Sci..

[41] Nordstrand T.M. (1995). Parametric study of the post-buckling strength of structural core sandwich panels. Compos. Struct..

[42] Nordstrand T. (2004). Analysis and testing of corrugated board panels into the post-buckling regime. Compos. Struct..

[43] Lu T.J., Chen C., Zhu G. (2001). Compressive behaviour of corrugated board panels. J. Compos. Mater..

[44] Garbowski T., Knitter-Piątkowska A. (2022). Analytical Determination of the Bending Stiffness of a Five-Layer Corrugated Cardboard with Imperfections. Materials.

[45] Mrówczyński D., Knitter-Piątkowska A., Garbowski T. (2022). Numerical Homogenization of Single-Walled Corrugated Board with Imperfections. Appl. Sci..

[46] Cillie J., Coetzee C. (2022). Experimental and Numerical Investigation of the In-Plane Compression of Corrugated Paperboard Panels. Math. Comput. Appl..

[47] Abaqus Unified FEA Software. https://www.3ds.com/products-services/simulia/products/abaqus.

[48] Czechowski L., Kmita-Fudalej G., Szewczyk W., Gralewski J., Bienkowska M. (2021). Numerical and Experimental Study of Five-Layer Non-Symmetrical Paperboard Panel Stiffness. Materials.

